# Atmospheric Pressure Pulsed Plasma Induces Cell Death in Photosynthetic Organs via Intracellularly Generated ROS

**DOI:** 10.1038/s41598-017-00480-6

**Published:** 2017-04-03

**Authors:** You-bin Seol, Jaewook Kim, Se-hong Park, Hong Young Chang

**Affiliations:** 1 Department of Physics, Korea Advanced Institute of Science and Technology, 373-1, Guseong-dong, Yuseong-gu, Daejeon 305-701 Republic of Korea; 2 Department of Biological Sciences, Korea Advanced Institute of Science and Technology, 373-1, Guseong-dong, Yuseong-gu, Daejeon 305-701 Republic of Korea; 3 Department of Electrical Engineering, Korea Advanced Institute of Science and Technology, 373-1, Guseong-dong, Yuseong-gu, Daejeon 305-701 Republic of Korea

## Abstract

The toxicity of atmospheric-pressure pulsed plasma on plant leaf tissues is studied. A nanosecond-pulsed plasma jet is applied to Arabidopsis thaliana leaves. In case of cotyledon, cell death is induced by treatment of only a few seconds. Cell death is also induced in the adult leaf by only 5 seconds of plasma treatment. Plasma induced reactive oxygen species (ROS) accumulation across the tissues within plasma-treated area. Plasma also induced direct physical damage to epidermis tissue of treated area but merely no damage to mesophyll. Thus, we propose direct physical damage in epidermis and ROS accumulation across the treated area induced cell death by plasma treatment. Plasma treatment with same duration in different organ also induced ROS accumulation but not plant death, suggests damage on photosynthetic organ by oxidative stress might be direct reason to induce cell death. We could also observe similar plasma induced death in *Solanum esculentum*, *Petunia axillaris*, and *Nicotiana benthamiana* but death is induced only in treated area. Thus, we propose atmospheric plasma induce oxidative stress in photosynthetic organ to induce cell death in plants.

## Introduction

Effect of plasma treatment on living organism has been widely studied in recent years, many focusing on its sterilization effect. Studies indicated that many microbial life forms underwent apoptosis or necrosis after only weak plasma treatment^1–9^ due to DNA damage and reactive oxygen species (ROS), which induce intracellular damage and subsequent cell death^7, 10^. However, eukaryotes were found to be less sensitive to plasma treatment^11–13^. This suggests that the dose of the plasma treatment safe for eukaryotic cells is toxic for prokaryotic cells and induces sterilization of bacteria^11–14^.

Studies about the effects of plasma treatment on biological samples concentrated on animal cells and prokaryotes, but a few groups studied the sterilization effects on food materials, e.g., sterilization of *Salmonella enterica* on fruits^15, 16^. In addition, plasma sterilization of grains contaminated with *Aspergillus* and *Penicillium* species was reported^17^. Some studies on in-package treatment of strawberries and cherry tomatoes indicated mildly negative effect on the fruit itself^18–20^.

Plasma treatment of plant material focuses on germination acceleration, disease resistance, and petal effects (also known as Lotus effect). Plasma-treated seeds of *Glycine max* showed increased vigor index and germination potential^21^. Same effect was observed, plasma treated seeds of *Solanum lycopersicum* showed increased germination rate and increased resistance to disease^18^. Furthermore, the diseased areas induced by natural infection in Emerald green plant leaves were cured by plasma treatment^11^. *Phaseolus vulgaris* treated by plasma in whole seeds showed increased germination rate, but when plasma was applied to the cotyledon tissue of the embryo of *Ph. vulgaris*, the petal effect diminished^22^.

Previous studies on biological systems claimed that plasma treatment induced DNA damage and ROS production intracellular-manner. Human fibroblast treated with plasma showed DNA damage mainly by single strand break and band damage^23^. 293 T cell treated with plasma also showed DNA damage and activation of the apoptosis pathway^24^, together with induction of intracellular ROS production. Moreover, 293 T cell, A549 cell, human bladder cancer cell and BEAS-2B cell was shown to induce intracellular ROS in response to plasma treatment^25^. Recent report on carrot callus tissue also confirmed intracellular ROS generation by plasma treatment is common trait, which can be observed throughout the plant kingdom, as well^26^.

In this study, we asked how photosynthetic organs react to plasma treatment. As expected, intracellular ROS was generated by plasma treatment. In our conditions, photosynthetic organs like cotyledons and adult leaves generated ROS in response to plasma exposure even for a very short time. Plant exposed to atmospheric plasma had direct damage in epidermis tissue, without severe damage in chloroplast in mesophyll. However, atmospheric plasma treated in photosynthetic organ induced cell death with chloroplasts damage, suggesting oxidative stress induced by ROS generated by plasma induced plant death.

## Results

### Very short exposure to plasma induces cell death and chlorophyll degradation via oxidative stress and direct damage in *A. thaliana*

**Figure 1:**
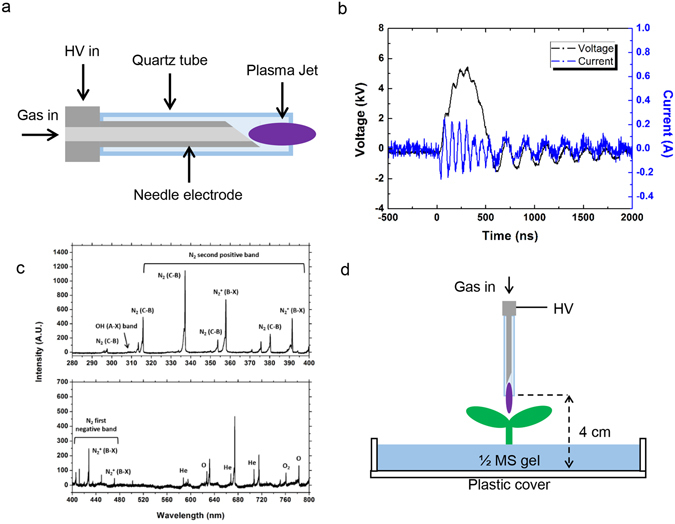
The experimental settings of the plasma treatment. (**a**) Structure of the plasma jet device (**b**) pulse voltage & discharge current signals of the plasma. (**c**) Emission spectra of the plasma. (**d**) Experimental setup of the plasma jet and the plant.

**Figure 2:**
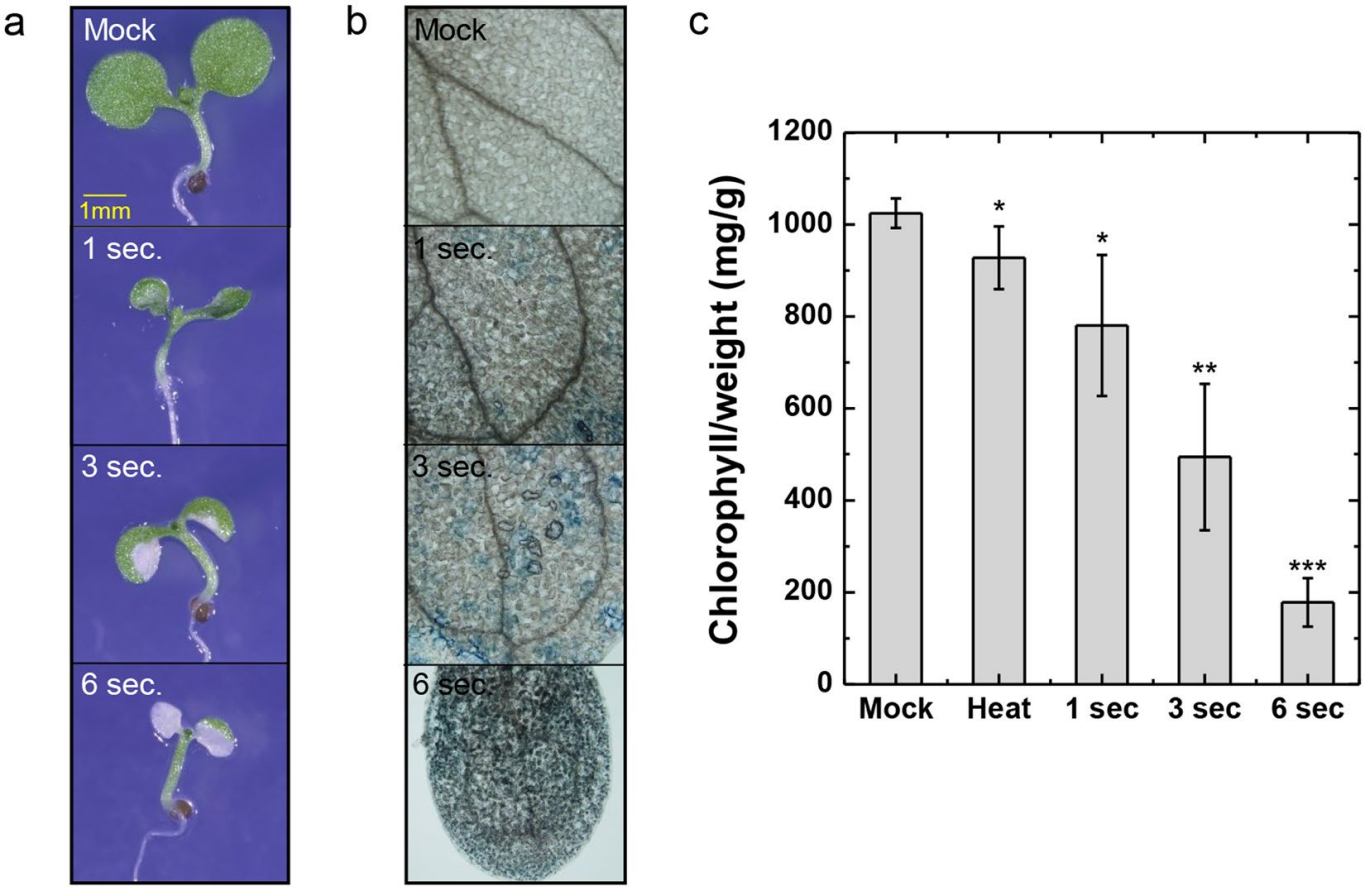
Arabidopsis seedlings treated with plasma on cotyledon induces cell death. (**a**) Phenotype of indicated treatment after 3 days incubation in long day condition. Scale bar = 1 mm. (**b**) Trypan blue staining of cotyledon of indicated treatment. (**c**) Total chlorophyll level of seedlings from indicated conditions. 1 sec, 3 sec, and 6 sec = plasma treatment for indicated time. Error bar = SD. Student’s t-test were used to indicate significant difference from Mock treatment, * < 0.05, ** < 0.01, *** < 0.001.

We exposed cotyledon to plasma, which was described as Fig. 1, for 1, 3, and 6 s, and observed seedling phenotype after 3 days after the plasma treatment (Fig. 2a). Plasma treated cotyledon was smaller and bleached area was increased by increasing plasma treatment duration (Fig. 2a). To analyze cotyledon phenotype was a result of cell death, we performed trypan blue staining and observed cotyledon area (Fig. 2b). As expected, plasma induced cell death in cotyledon tissue (Fig. 2b). When we measure chlorophyll concentration, plasma treatment resulted in decreased chlorophyll concentration per fresh weight by plasma treatment dose-dependent manner (Fig. 2c). In conclusion, atmospheric plasma treatment to cotyledon induced cell death and chlorophyll degradation.

**Figure 3:**
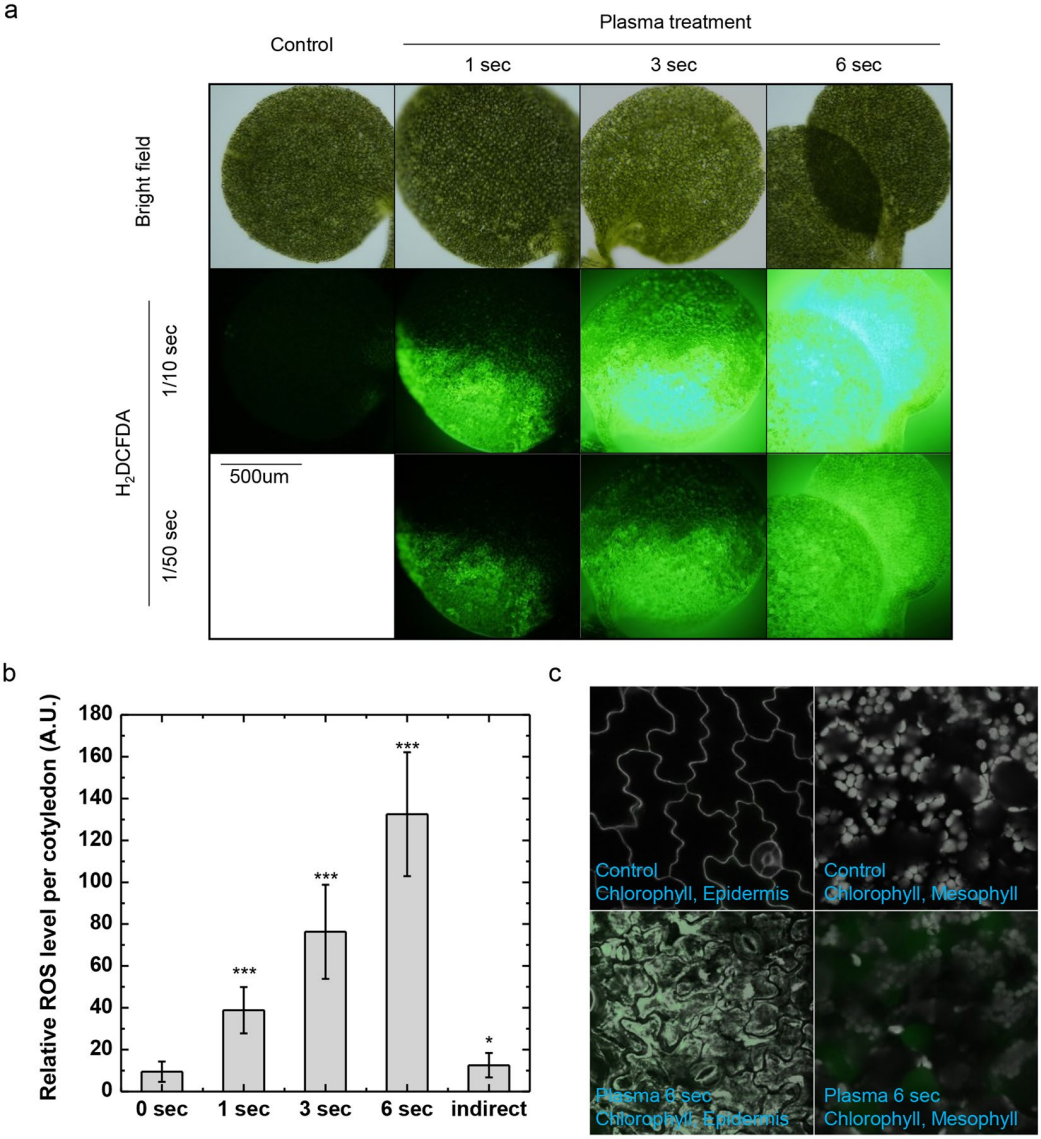
Atmospheric plasma induced ROS accumulation across the treated area in cotyledon. (**a**) DCFDA fluorescence image of cotyledon treated with plasma for indicated times (s). Representative image were determined from 5 experiments for each conditions. (**b**) Relative ROS level was measured in direct plasma treated seedlings and indirectly treated samples. To measure ROS level, we used 20 cotyledons treated with indicated conditions and DCFDA signal intensity were measured with same exposure time taken fluorescence image data. Signal was measured for whole cotyledon as mean grey value. Error bar = SD. Student’s t-test were used to indicate significant difference from Mock treatment, * < 0.05, ** < 0.01, *** < 0.001. (**c**) DCFDA fluorescence image of cotyledon treated with 6 seconds of plasma or not by confocal microscope. Grey signals in epidermis images are propidium iodide fluorescence to visualize cell walls and nucleus, and grey signals in mesophyll images are chlorophyll auto-fluorescence to visualize relative plastid location. Representative image were determined from 5 experiments for each conditions.

**Figure 4:**
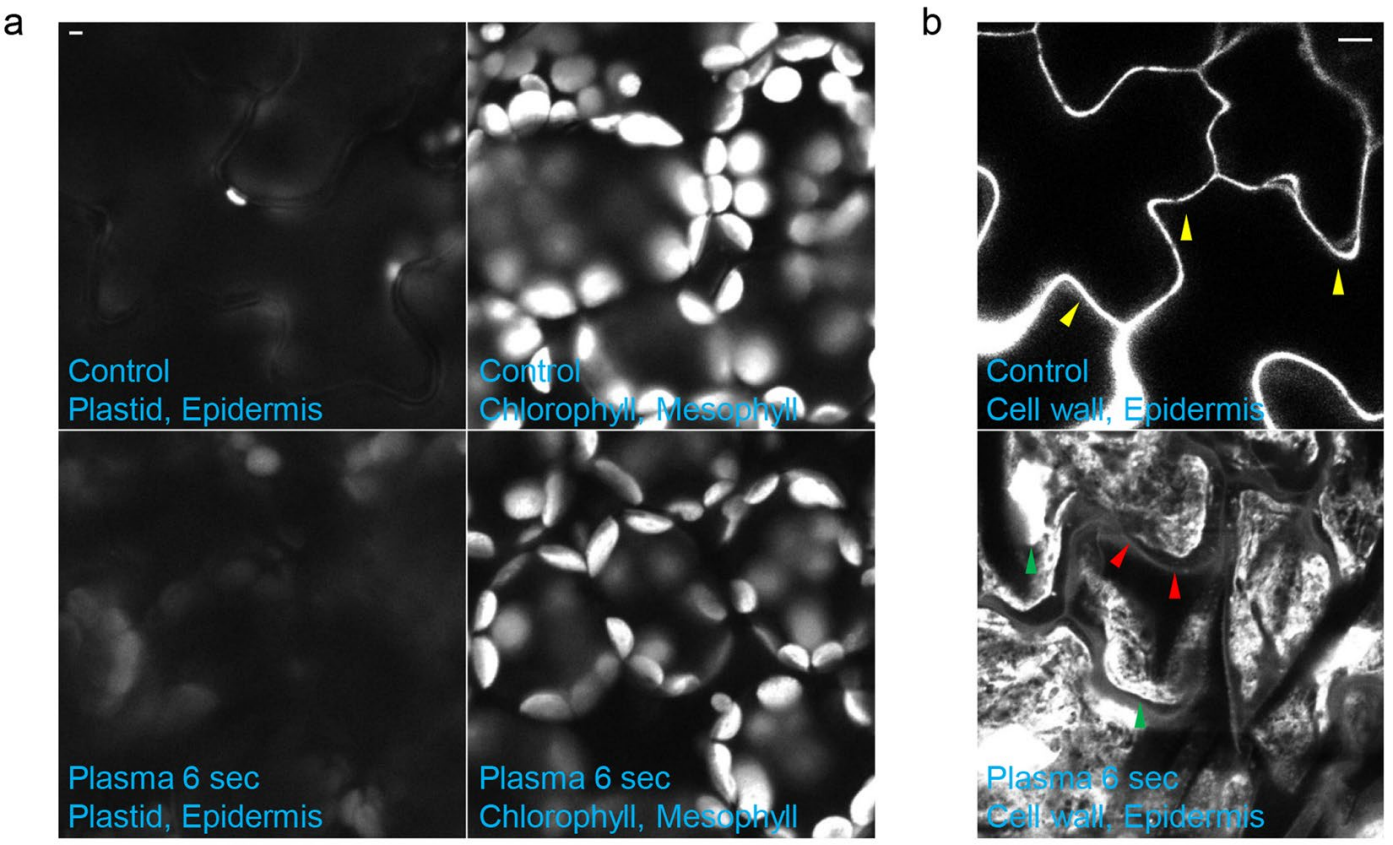
Epidermis layer is directly damaged through plasma treatment. (**a**) Chlorophyll auto-fluorescence image of cotyledon treated with 6 seconds of plasma or not by confocal microscope. Scale bar = 2 um. Representative image were determined from 5 experiments for each conditions. (**b**) Cell wall fluorescence image of cotyledon epidermis treated with 6 seconds of plasma or not by confocal microscope. Yellow arrowheads indicate normal cell wall signal by propidium iodide, green arrowheads indicate unknown intracellular stained propidium iodide signal, and red arrowheads indicates weak residual cell wall signal stained by propidium iodide. Representative image were determined from 5 experiments for each conditions. Scale bar = 2 um.

Plasma was previously identified as ROS-inducer in other organisms, which is a reason of cytotoxicity to many organisms when it is accumulated more than ROS-scavenger capacity. Plasma treatment only with few seconds might induce excessive ROS in plant and induce oxidative stress enough to induce cell death. To test this hypothesis, we performed DCFDA staining in plasma treated seedlings and obtained fluorescent signal (Fig. 3a). Plasma induced stronger DCFDA signal in treated area of cotyledon in plasma dose-dependent manner (Fig. 3a,b). Then, we asked which tissue accumulates ROS triggered by plasma treatment (Fig. 3c). Compare to control cotyledon, DCFDA signal was detected in every tissues we observed. Surprisingly, DCFDA signal was high throughout cells of epidermis and concentrated in certain areas in mesophyll (Fig. 3c). In addition, we observed cell structure in epidermis has interrupted by plasma but no significant difference in mesophyll (Fig. 3c). These data suggest plasma induce direct damage in epidermis and induce ROS accumulation throughout the treated area. Indeed, plastid in cotyledon epidermis was not detected in plasma treated samples, indicates plasma induced direct damage disrupted plastids in epidermis (Fig. 4a). However, plastid auto-fluorescence signal in mesophyll was similar in both plasma treated and control seedlings (Fig. 4a), indicating plasma induced direct damage is introduced mainly in epidermis tissue. To observe damage in epidermis layer in detail, we detected propidium iodide (PI) signal, which is mainly staining cell walls and nuclei (Fig. 4b). Compare to control signals, PI signal in plasma treated sample was largely disrupted (Fig. 4b). In summary, plasma treatment induced direct damage mainly in epidermis.

### Plasma treatment on photosynthetic organs induce cell death in *A. thaliana*

DCFDA detects H_2_O_2_ majorly^27–29^, thus at least our plasma treatment induced rapid ROS accumulation including H_2_O_2_. Rapid accumulation of H_2_O_2_ results in oxidative stress to photosynthetic organs and triggers cell death^30–32^. However, plasma induces other aspects, which might induce cytotoxicity, such as heat, pressure, electric field, and ultra-violet (uv) light. To identify these effect of these factors, we treated 37 °C heat for 10 secs or He gas pressure for 10 secs or plasma treatment on quarts slide-glass covered cotyledon (indirect) for 6 secs and performed DCFDA staining (Supplemental Figure 1a). Compare to plasma treated samples, those heat-, air pressure-, and indirect- treated samples stained very weak for DCFDA staining (Supplemental Figure 1a). Thus, these conditions represent stress conditions other than oxidative stress. To identify whether these factors induce direct damage, we stained PI to those heat-, air pressure-, and indirect- treated samples and epidermis PI signal was similar with control, indicating these factors are not direct reason for epidermis damage induced by plasma (Supplemental Figure 1b). Consistently, heat-, air pressure-, and indirect- treated samples showed normal phenotype (Supplemental Figure 1c). In conclusion, at least those factors are not direct reasons to induce plasma induced cell death or ROS or epidermis damage.

**Figure 5:**
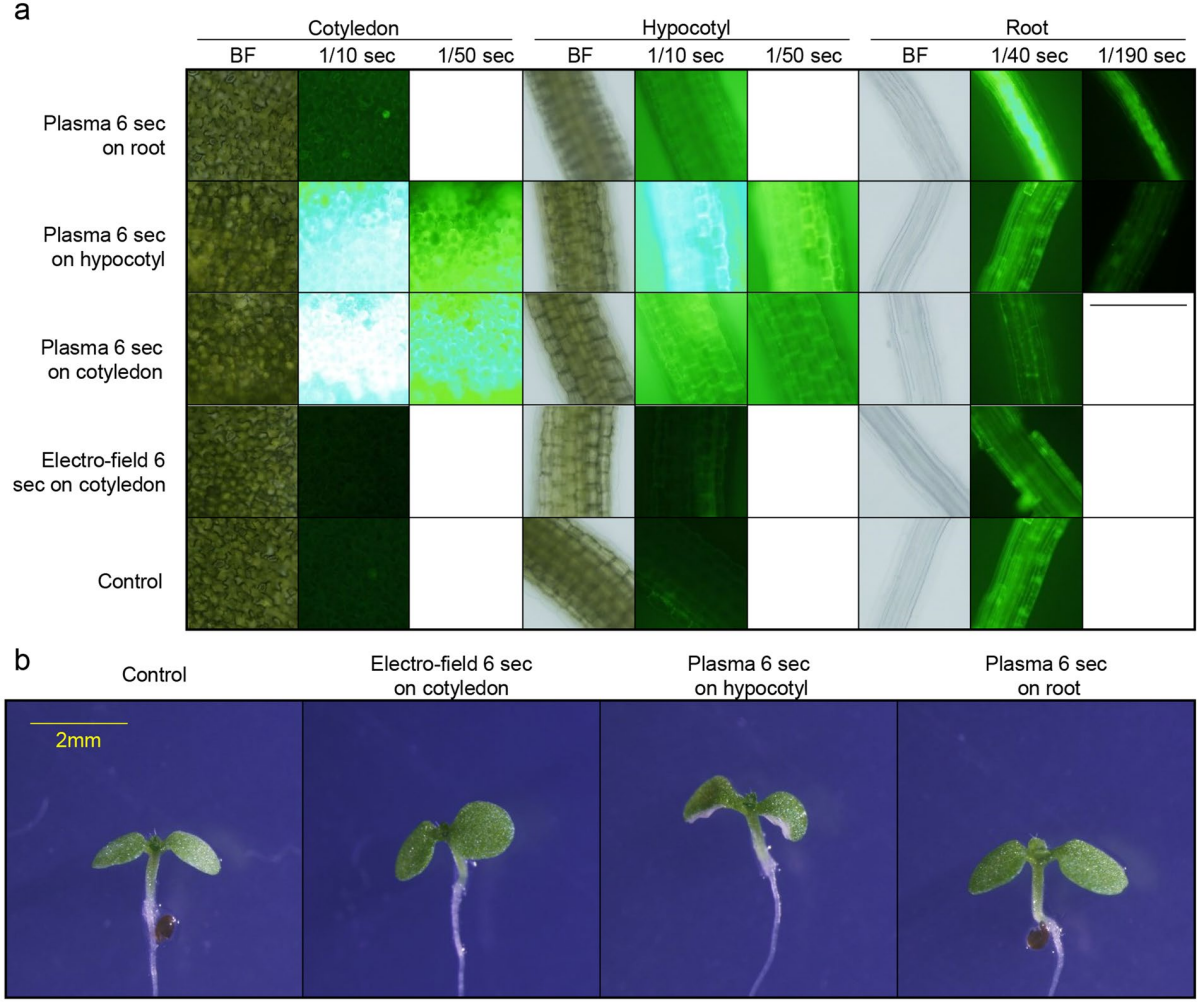
Photosynthetic organ treated plasma induces cell death. (**a**) Plasma treated in different organs for 6 seconds. BF indicates bright field. Time indication under indicated tissue is exposure time (seconds). Scale bar = 500 um. Representative image were determined from 5 experiments for each conditions. (**b**) Phenotype of seedlings treated with indicated conditions after 3 days of incubation in long day. Scale bar = 2 mm. Representative image were determined from 5 experiments for each conditions.

We then asked whether plasma treatment to other tissues could induce plant death. To verify plasma treatment induces ROS accumulation in any tissue, we detected DCFDA signal in seedlings with plasma treated on different tissues (Fig. 5a). While considering tissue-specific plasma response, we also considered electric field treated on cotyledon seedlings. As expected, plasma treatment introduced ROS accumulation in treated area (Fig. 5a) while hypocotyl treated sample also accumulated ROS in cotyledon due to the size of our plasma source. On the other hand, electric field treatment on cotyledon did not induce ROS accumulation (Fig. 5a). Consistently, plasma treatment on root, hypocotyl and electric field treatment on cotyledon did not induce plant death (Fig. 5b). In conclusion, plasma treatment on cotyledon induce oxidative stress and cell death.

**Figure 6:**
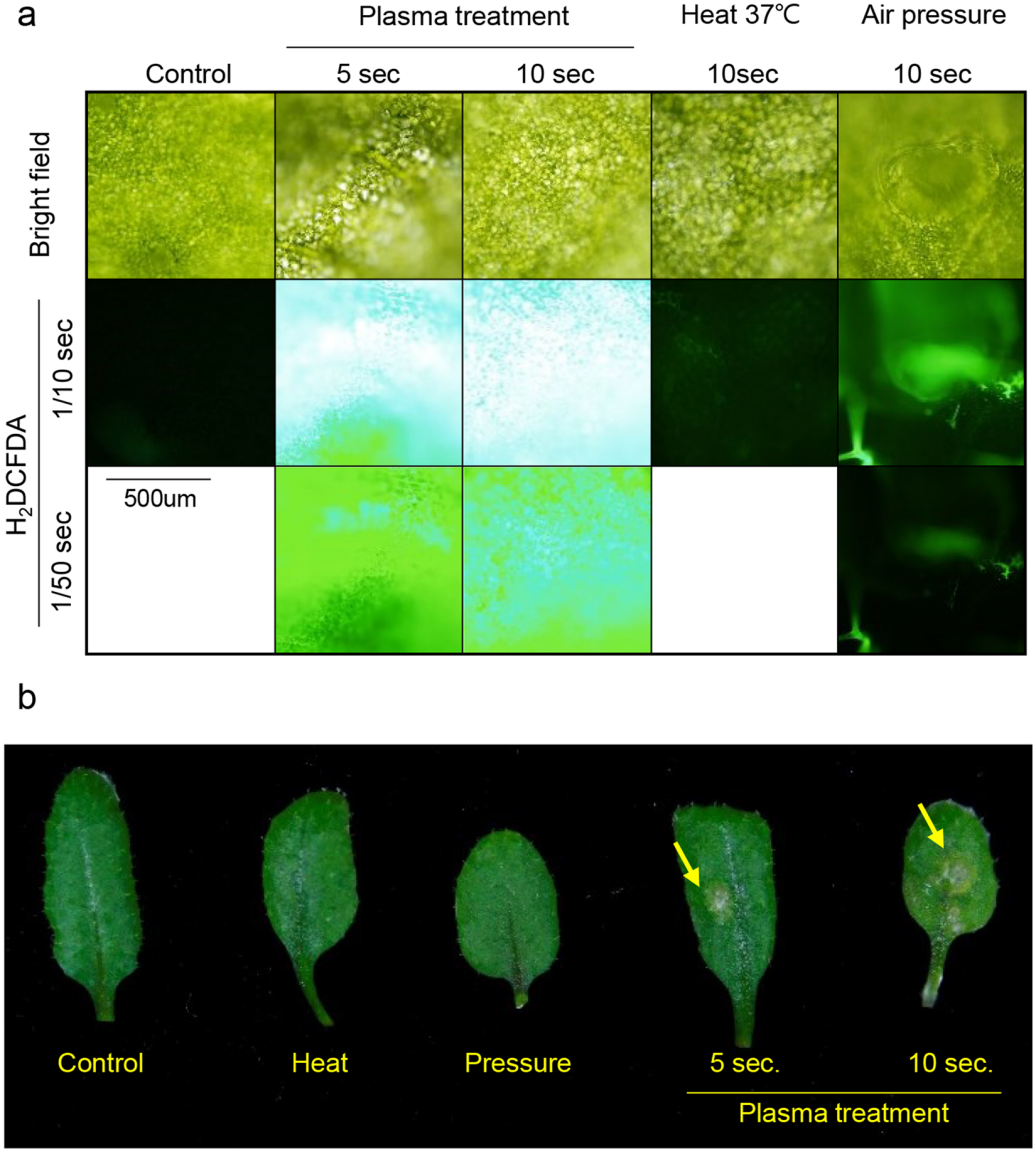
Adult leaf treated with plasma. (**a**) DCFDA fluorescence image of adult leaf in treated area. Upper panel indicates treatments. Times indicated under H_2_DCFDA is exposure time (seconds). Scale bar = 500 um. Representative image were determined from 5 experiments for each conditions. (**b**) Phenotype of adult leaf after plasma treatment in long day condition for 3 days. Yellow arrow indicates plasma treated area. Heat and Pressure indicates 37 °C heat for 10 seconds and air pressure for 10 seconds, each.

Plasma induced oxidative stress could induce plant death only in cotyledon, which indicates photosynthetic apparatus damage induces cell death. To prove this hypothesis, we treated plasma on adult leaf and observed ROS accumulation and following cell death (Fig. 6). As in cotyledon, plasma also induced ROS accumulation in dose dependent manner in adult leaf (Fig. 6a). This ROS accumulation was followed by bleaching phenotype in treated area (Fig. 6b), indicating plasma treatment on adult leaf induces similar physiological response as in cotyledon.

### Plasma treatment on photosynthetic organs induce cell death in Planta

**Figure 7:**
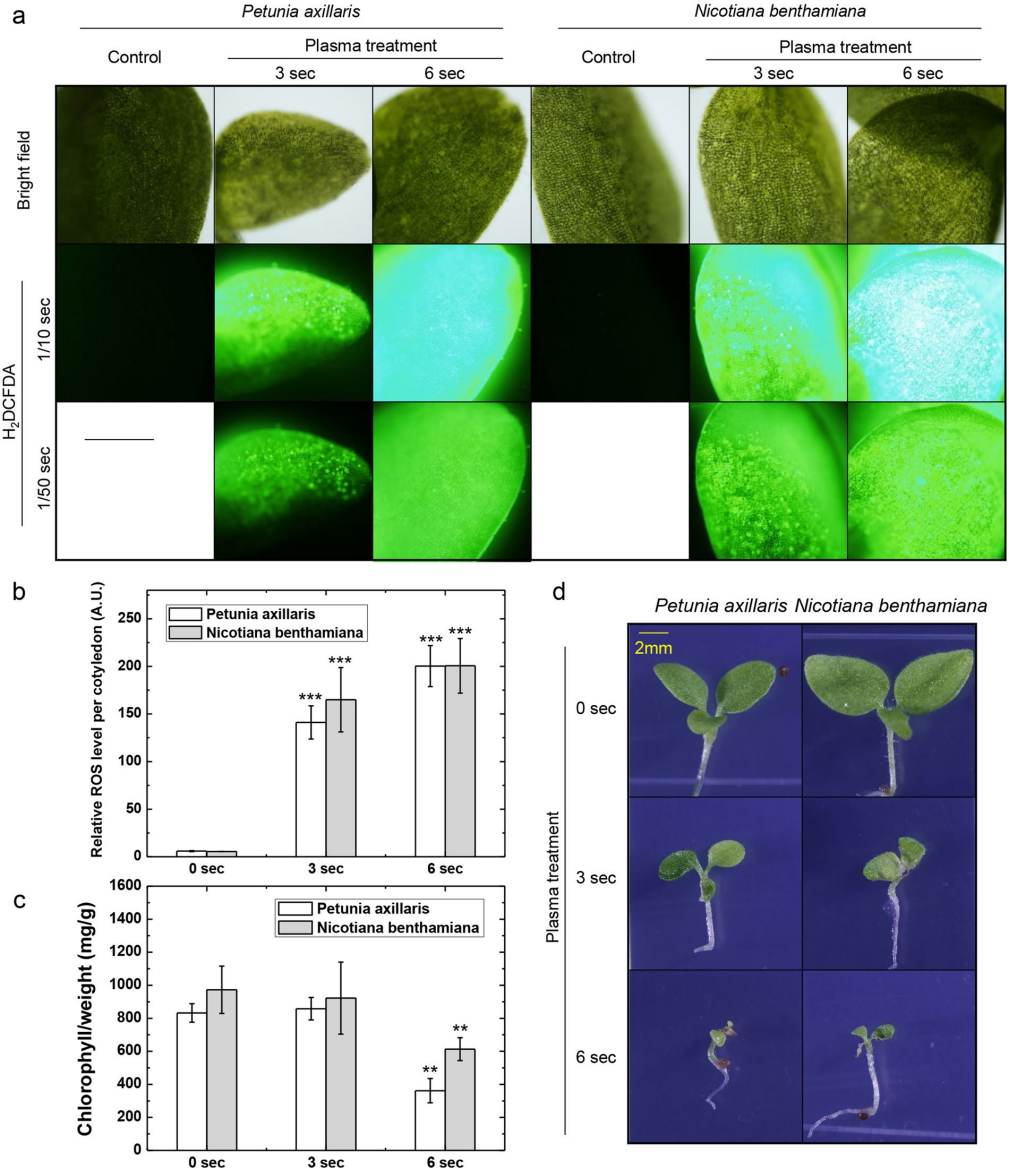
Petunia and tobacco seedlings treated with plasma accumulates ROS and induces cell death. (**a**) DCFDA image of plasma treated petunia and tobacco right after plasma treatment. Time indication under H_2_DCFDA indicates exposure time (sec.) Scale bar = 500 um. Representative image were determined from 5 experiments for each conditions. (**b**) Relative ROS level was measured in direct plasma treated seedlings and indirectly treated samples. To measure ROS level, we used 5 cotyledons treated with indicated conditions and DCFDA signal intensity were measured with same exposure time taken fluorescence image data. Signal was measured for whole cotyledon as mean grey value. Error bar = SD Student’s t-test were used to indicate significant difference from Mock treatment of each plant, * < 0.05, ** < 0.01, *** < 0.001. (**c**) Total chlorophyll level from samples after 5 days. 0 s, 3 s, and 6 s indicates plasma treatment time (seconds). Error bar = SD. Student’s t-test were used to indicate significant difference from Mock treatment of each plant, * < 0.05, ** < 0.01, *** < 0.001. (**d**) Phenotype of plasma treated seedlings after 5 days of plasma treatment in long-day incubation. Scale bar = 2 mm. Representative image were determined from 5 experiments for each conditions.

We wanted to further study whether our finding is general to plants, we treated plasma on cotyledons of *P. axillaris* and *N. benthamiana* seedlings (Fig. 7). As in *Arabidopsis* cotyledon, plasma treatment induced ROS accumulation in *P. axillaris* and *N. benthamiana* cotyledon in dose-dependent manner (Fig. 7a,b). Although chlorophyll was decreased in slower kinetics, chlorophyll concentration per fresh weight was lowered by plasma treatment in these species (Fig. 7c). Consistently, plasma induced plant death in seedlings of these species as in *Arabidopsis*. To further identify adult leaf response in other plant system, we treated plasma on adult leaves of *S. esculentum* (Cv. Micro tom) and *P. axillaris* and observed phenotype (Supplemental Figure 2). As expected plasma induced cell death in treated area of adult leaves in these species (Supplemental Figure 2).

**Figure 8:**
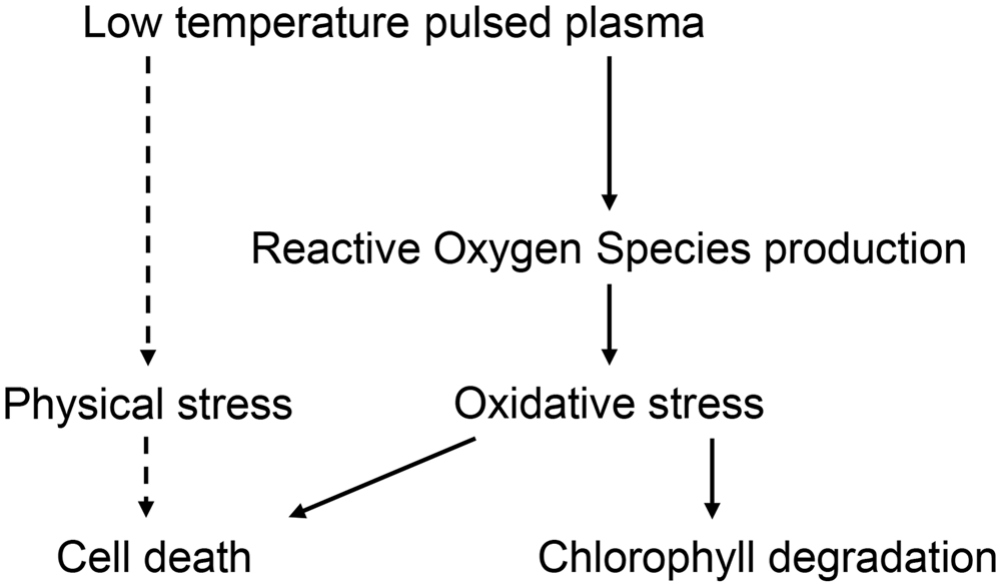
Proposed model for induced cell death in photosynthetic organs by plasma treatment. Low temperature pulsed plasma induce ROS accumulation in every tissue (solid arrow) and direct damage in epidermis (dotted arrow). ROS accumulation induce cell death and chlorophyll disruption, while direct damage in epidermis induce epidermis cell to be disrupted and ROS leaked from epidermis might contribute to ROS accumulation in mesophyll.

To summarize, atmospheric pulsed plasma treatment on photosynthetic organs such as cotyledons or adult leaves induce ROS accumulation or direct physical stress only on epidermis. Oxidative stress induced by ROS accumulation induce chloroplast damage and cell death, which can be observed in plant species (Fig. 8).

## Discussion

Various plasma components can interact with biological systems^33^. First, high electric field can have an effect. In this experiment, the maximum electric field applied to the plant cell was over 1 kV/cm, which was enough for cell permeabilization^34, 35^. It was reported that ultraviolet light was another component of plasma treatment^33^. In optical emission spectrometry (OES) tests, there were strong UV signals within a wide range. Comparing to indirect treatment results (Supplemental Figure 1), we have confirmed that the above two components of the plasma treatment had no significant effects on the plant cell. The direct current can also damage the cell because our plasma device is a single electrode jet^36^. Using a dielectric block, we checked the effect of electric current on the sample and no significant effect was observed. Finally, the ROS were the most important factor in plasma treatment. It was reported that they induced cell damage via direct physical and indirect cellular chemical interactions^33^. Again in OES and cellular fluorescence tests, we found a high amount of ROS produced by the plasma, which is the main contributor of plasma adverse effects on the plants.

We applied low temperature plasma to study plasma effect on plants and discovered plasma treatment on photosynthetic organs induced ROS accumulation and direct damage to induce cell death. We could observe plasma induce direct physical damage on epidermis layer specifically. Previous reports indicate high electric field induce permeabilization^34, 35^, thus plasma-induced cell structure disruption also induce micro-pores in cell periphery. Thus, ROS generated in epidermis layer might leaked into mesophyll layer, which is indirectly evidenced by DCFDA signal in cavity area of mesophyll. Thus epidermal cell disruption is one of the reason of ROS accumulation in mesophyll.

Our result indicates toxic effect of plasma on photosynthetic organs. Since atmospheric plasma is relatively easy to handle, this system implies application as pesticide. In addition, pathogen-infected area can be eliminated by atmospheric plasma to prevent further infection with providing unnecessary wound stress for plants. Thus, even though we found plasma could be toxic element to planta, we can use this system to protect our crop plants while preventing ourselves.

## Methods and Materials

### Atmospheric-Pressure Plasma setting

A needle-shaped plasma jet was used for the treatment (Fig. 1a). The device consisted of a 4 cm long stainless-steel needle and a 4.3 cm long quartz tube. The inner diameter of the quartz tube was 2 mm, and the outer diameter was 3 mm. The feeding gas, He at a flow rate of 2 slm, flew through the needle, and the high voltage needle generated a jet plume.

The device was operated by a home-made HV (high voltage) nanosecond pulse generator (CNSL, KAIST) with a peak voltage of 5 kV, pulse width of half-maximum 350 ns with a repetition rate of 20 kHz, and the average power of 1 W (Fig. 1b). It provided an operation temperature below 40 °C, which prevented thermal damage to the biological target. The plasma voltage and current signals were measured by an HV probe and current probe (Tektronix) connected with an oscilloscope (Lecroy 104Xi).

Plasma-produced reactive species were observed by optical emission spectroscopy using a monochromator (DONGWOO OPTRON) and an ICCD camera (iStar, Andor) (Fig. 1c). Strong nitrogen lines were observed with He and reactive oxygen species, indicating the presence of He metastables, which interact with ambient air in plasma discharge. In addition, the OH 309 nm band was observed, whose intensity was much weaker than other species. No nitrogen oxide band was observed^37–39^.

The plant treatment is illustrated in Fig. 1d. The plant agar plate is on the ground and the plasma jet is generated on the plant directly. The distance between the electrode and the ground is 4 cm and the average distance between the electrode and the plant sample is 2.5 cm. In case of indirect treatment, cover glass is placed on the plant sample to prevent direct exposure to the plasma.

### Plant material and growth conditions

*Arabidopsis thaliana*, *Nicotiana benthamiana*, *Petunia axillaris*, and *Solanum esculentum* (cv. Micro Tom) were grown in a 16 h light/ 8 h dark cycle at 22 °C for general growth and seed harvesting.

*A. thaliana* seedlings (Col-0) were grown under long-day condition for 3 days, whereas other species were grown for 5 days, and treated with plasma for the indicated lengths of time. Adult leaves of *A. thaliana* were harvested from 28-day old plants, and those of *S. esculentum* and *P. axillaris* from 45-day old plants. *A. thaliana* adult leaf was taken within number 5–6, and *S. esculentum* and *P. axillaris* leaf was taken within number 7–9 leaf.

To introduce ‘indirect’ treatment of plasma, MARIENFELD Microscope Cover Glasses thinkness No. 1 (Lauda-Königshofen, Germany) was used to directly cover the cotyledon and we treated plasma.

### DCFDA staining

Tissues to observe the ROS signals were submerged in 10 µM 2′,7′–dichlorofluorescin diacetate (DCFDA, SIGMA-Aldrich, D6883-50MG, Darmstadt, Germany) solution in 10 mM Tris-Cl buffer (pH 7.4) for 10 min. The samples were briefly washed and mounted with distilled water. An Olympus BX21 model was used to obtain the DCFDA signal. Seedling number to measure DCFDA signal intensity and concluding were indicated in each figure legends.

### Trypan blue staining

Trypan blue staining protocol of ours is previously reported protocol^40^. Samples were submerged in trypan blue working solution (10 g phenol, 10 ml glycerol, 10 ml lactic acid, 10 ml water, and 0.02 g of trypan blue in 30 ml trypan blue working solution) and boiled for 1 min. We used BIONEER C-9015 model from Daejeon, Korea for phenol, JUNSEI 27210S0350 for glycerol from Chuo-ku, Tokyo, Japan, SAMCHUN CHEMICALS L0017 for lactic acid from Gyung-gi, Korea, SIGMA-Aldrich T8657-500G for Trichloroacetic acid from Darmstadt, Germany, and SIGMA-Aldrich T6146-5G for trypan blue. Samples were then stained in trypan blue solution for 12 h at room temperature and rinsed twice with TCA solution (TriChloroAcetic acid : distilled water 3:2 v/v). Samples were mounted with TCA solution and observed with an Olympus BX21 model. We concluded after observing 3 seedlings in each condition.

### Chlorophyll measurement

Chlorophyll concentration per weight was measured as described previously with modifications^41^. Then, seedlings were submerged in 100% ethanol and incubated for 1 h at 4 °C under completely dark conditions in shaking incubator. Samples were then taken to measure absorbance on 648.6 and 664.2 nm. Chlorophyll A was determined by 5.2007A_648.2 nm_+13.5275A_664.2 nm_ (mg), and chlorophyll B was determined by 22.4327A_648.2 nm_−7.0741A_664.2 nm_ (mg). To normalize the value, we seedlings were weighed as fresh weight (weight right after harvesting), but for a prep, usually 3~5 seedlings were taken and 3 preps were done for analysis. Absorbance values were subdivided with fresh weight in each samples.

### Fluorescence microscopic imaging

Seedlings were mounted with distilled water and observed using either an epifluorescence microscope (Olympus BX-21) or a confocal microscope (Zeiss LSM 780). Cell walls and nuclei were visualized by staining with propidium iodide (PI, SIGMA, P4170-25MG, 30 μM in distilled water) for 10 minute and samples were mounted with ddw. To quantify DCFDA intensities, all fluorescence images were taken at the same exposure time. DCFDA signal intensities were quantified using ImageJ with mean grey value options by measuring data within whole cotyledon region. Numbers of cotyledon to measure the DCFDA signal intensity were indicated in each figure legends. PI signal was gained between 600–630 nm, and chlorophyll autofluorescence signal was gained between 600–780 nm.

## Electronic supplementary material

Supplementary Information
