# Supplementary material for: Atmospheric Pressure Pulsed Plasma Induces Cell Death in Photosynthetic Organs via Intracellularly Generated ROS
Source: Sci Rep. 2017 Apr 3;7:589. doi: 10.1038/s41598-017-00480-6 (PMC5428426; doi:10.1038/s41598-017-00480-6)
Supplement: Supplementary file 1 — Supplementary Information [file 41598_2017_480_MOESM1_ESM.pdf]

Supplementary Information for

Atmospheric Pressure Pulsed Plasma Induces Cell Death in Photosynthetic Organs via Intracellularly Generated ROS.

Youbin Seol[a], Jaewook Kim[b], Se-hong Park[c], and Hong Young Chang[a*]

[a] Department of Physics, Korea Advanced Institute of Science and Technology, 373-1, Guseong-dong, Yuseong-gu, Daejeon 305-701, Republic of Korea

[b] Department of Biological Sciences, Korea Advanced Institute of Science and Technology, 373-1, Guseong-dong, Yuseong-gu, Daejeon 305-701, Republic of Korea

[c] Department of Electrical Engineering, Korea Advanced Institute of Science and Technology, 373-1, Guseong-dong, Yuseong-gu, Daejeon 305-701, Republic of Korea

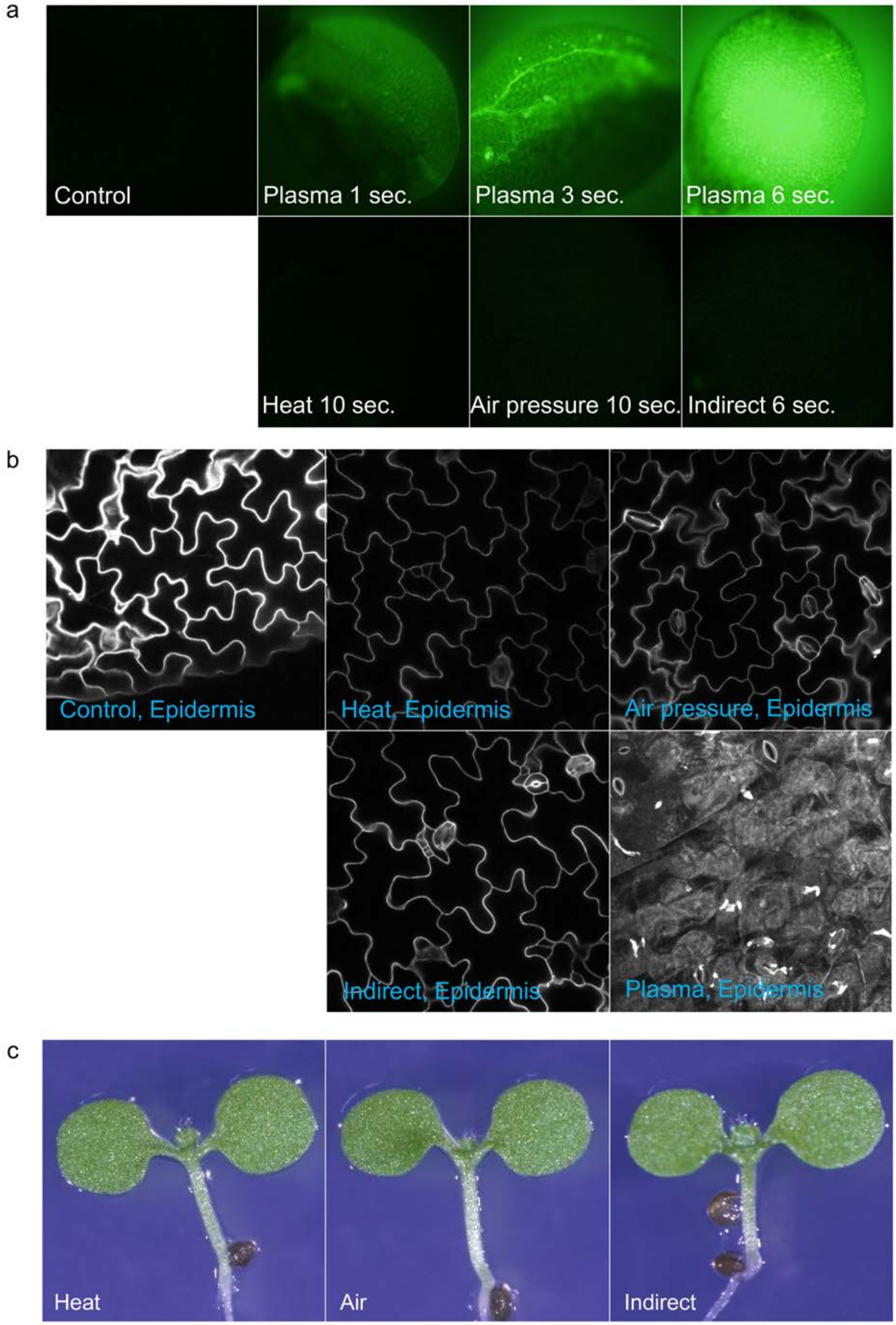
a
Control
Plasma 1 sec.
Plasma 3 sec.
Plasma 6 sec.
Heat 10 sec.
Air pressure 10 sec.
Indirect 6 sec.
b
Control, Epidermis
Heat, Epidermis
Air pressure, Epidermis
Indirect, Epidermis
Plasma, Epidermis
c
Heat
Air
Indirect

Supplementary figure S1. a. DCFDA fluorescence image of cotyledon treated by plasma or plasma components. Heat indicates 37°C heat, and indirect indicates plasma treatment with quartz slide cover on cotyledon. Representative image were determined from 5 experiments for each conditions. b. Epidermis cell wall image of cotyledon treated by plasma components. Heat indicates 37°C heat for 10 seconds, air pressure indicates gas pressure for 6 seconds, and indirect indicates plasma treatment with quartz slide cover on cotyledon for 6 seconds. Representative image were determined from 5 experiments for each conditions. c. Phenotype of seedlings treated with indicated conditions after 3 days of incubation in long day. Heat indicates 37°C heat for 10 seconds, air pressure indicates gas pressure for 6 seconds, and indirect indicates plasma treatment with quartz slide cover on cotyledon for 6 seconds. Representative image were determined from 5 experiments for each conditions.

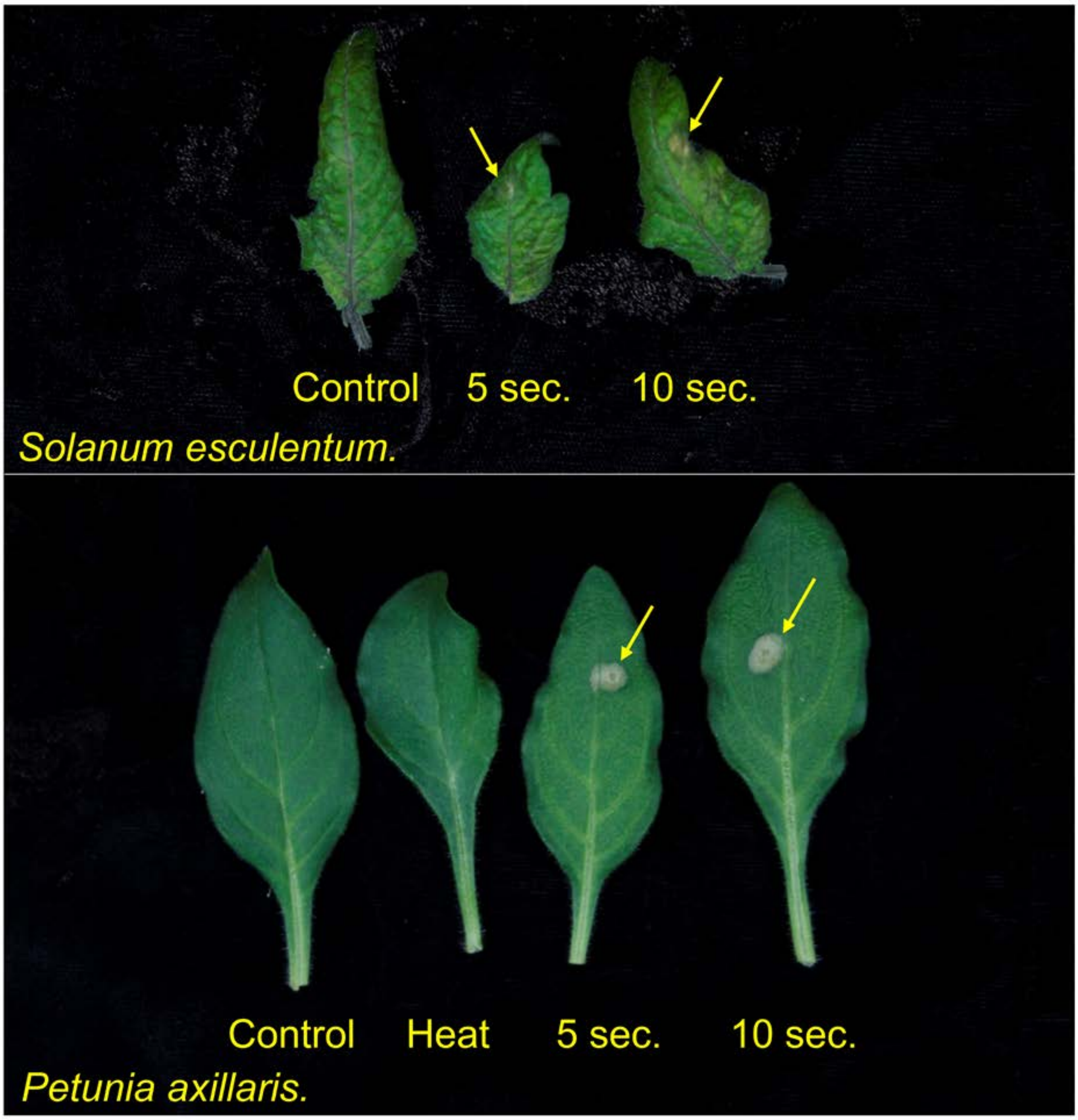

Supplementary figure S2. Adult leaf phenotype treated with plasma. Leaves of petunia and tomato was treated by plasma for indicated time (seconds) and incubated in long day for 3 days.
